# Association of liver dysfunction with outcomes after cardiac surgery—a meta-analysis

**DOI:** 10.1093/icvts/ivac280

**Published:** 2022-12-08

**Authors:** Hristo Kirov, Tulio Caldonazo, Katia Audisio, Mohamed Rahouma, N Bryce Robinson, Gianmarco Cancelli, Giovanni J Soletti, Michelle Demetres, Mudathir Ibrahim, Gloria Faerber, Mario Gaudino, Torsten Doenst

**Affiliations:** Department of Cardiothoracic Surgery, Friedrich-Schiller-University Jena, Germany; Department of Cardiothoracic Surgery, Friedrich-Schiller-University Jena, Germany; Department of Cardiothoracic Surgery at New York Presbyterian, Weill Cornell Medical Center, USA; Department of Cardiothoracic Surgery at New York Presbyterian, Weill Cornell Medical Center, USA; Department of Cardiothoracic Surgery at New York Presbyterian, Weill Cornell Medical Center, USA; Department of Cardiothoracic Surgery at New York Presbyterian, Weill Cornell Medical Center, USA; Department of Cardiothoracic Surgery at New York Presbyterian, Weill Cornell Medical Center, USA; Samuel J. Wood Library and C.V. Starr Biomedical Information Center, Weill Cornell Medicine, New York, NY, USA; Department of General Surgery, Maimonides Medical Center, Brooklyn, NY, USA; Nuffield Department of Surgical Sciences, University of Oxford, Oxford, UK; Department of Cardiothoracic Surgery, Friedrich-Schiller-University Jena, Germany; Department of Cardiothoracic Surgery at New York Presbyterian, Weill Cornell Medical Center, USA; Department of Cardiothoracic Surgery, Friedrich-Schiller-University Jena, Germany

**Keywords:** Liver dysfunction, MELD score, Child–Turcotte–Pugh score, Open heart surgery

## Abstract

**OBJECTIVES:**

The aim of this study was to perform a meta-analysis of studies reporting outcomes in patients with liver dysfunction addressed by the model of end-stage liver disease and Child–Turcotte–Pugh scores undergoing cardiac surgery.

**METHODS:**

A systematic literature search was conducted to identify contemporary studies reporting short- and long-term outcomes in patients with liver dysfunction compared to patients with no or mild liver dysfunction undergoing cardiac surgery (stratified in high and low score group based on the study cut-offs). Primary outcome was perioperative mortality. Secondary outcomes were perioperative neurological events, prolonged ventilation, sepsis, bleeding and/or need for transfusion, acute kidney injury and long-term mortality.

**RESULTS:**

A total of 33 studies with 48 891 patients were included. Compared with the low score group, being in the high score group was associated with significantly higher risk of perioperative mortality [odds ratio (OR) 3.72, 95% confidence interval (CI) 2.75–5.03, *P* < 0.001]. High score group was also associated with a significantly higher rate of perioperative neurological events (OR 1.49, 95% CI 1.30–1.71, *P* < 0.001), prolonged ventilation (OR 2.45, 95% CI 1.94–3.09, *P* < 0.001), sepsis (OR 3.88, 95% CI 2.07–7.26, *P* < 0.001), bleeding and/or need for transfusion (OR 1.95, 95% CI 1.43–2.64, *P* < 0.001), acute kidney injury (OR 3.84, 95% CI 2.12–6.98, *P* < 0.001) and long-term mortality (incidence risk ratio 1.29, 95% CI 1.14–1.46, *P* < 0.001)

**CONCLUSIONS:**

The analysis suggests that liver dysfunction in patients undergoing cardiac surgery is independently associated with higher risk of short and long-term mortality and also with an increased occurrence of various perioperative adverse events.

**Figure:**
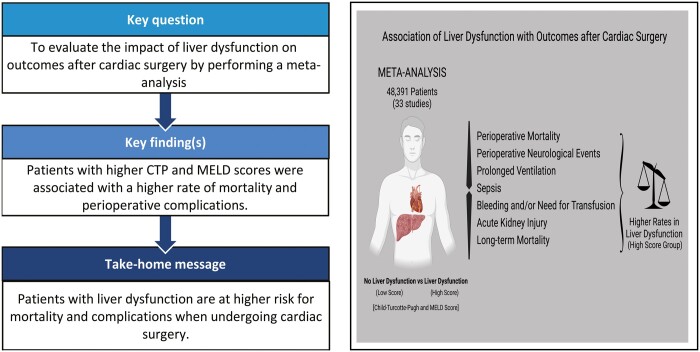

## INTRODUCTION

The evaluation of various preoperative risk factors and their association with surgical outcome has become an important aspect of clinical decision-making. In cardiac surgery, large databases have been employed to develop numerous risk scores with the aim to accurately predict surgical risks [1, 2], provide information for patient counselling, assist with evaluation of surgical quality or be used for differentiated financial reimbursement [3].

Liver dysfunction has been recognized early as a critical risk factor [4]. As a result, patients with liver dysfunction are rarely included in these databases, presumably because surgeons have understood this risk and abstained from routinely operating on these patients. With a changing and ageing population, the number of patients with evidence of liver dysfunction has increased in recent years [5, 6] and results suggest that perioperative risks are not appropriately assessed in these cases using the classical risk scores [7, 8].

A thorough assessment of liver dysfunction on mortality and other perioperative complications has not been published, yet. Thus, the only current way to evaluate the impact of liver dysfunction in cardiac surgery in a large number of patients is to evaluate and summarize the existing body of scientific publications. We therefore set out to systematically review the literature and thoroughly assess the effect of liver dysfunction by way of a meta-analysis on clinical outcome after cardiac surgery, with a focus on short- and long-term mortality and all-classic adverse events during the perioperative period.

## METHODS

Ethical and IRB approval of this analysis were not required as no human or animal subjects were involved. This review was registered with the National Institute for Health Research International Registry of Systematic Reviews (PROSPERO, CRD42021239043).

### Search strategy

A medical librarian (Michelle Demetres) performed a comprehensive literature search to identify contemporary studies reporting short- and long-term outcomes in patients with liver dysfunction compared to patients with no or mild liver dysfunction undergoing open heart surgery. Searches were run on 24 February 2021 in the following databases: Ovid MEDLINE^®^ (ALL; 1946 to present); Ovid EMBASE (1974 to present); and The Cochrane Library (Wiley). The search strategy for Ovid MEDLINE is available in Supplementary Material, Table S1.

### Study selection and data extraction

The study selection followed the Preferred Reporting Items for Systematic Reviews and Meta-Analyses (PRISMA) strategy. After de-duplication, records were screened by 2 independent reviewers (Tulio Caldonazo and Hristo Kirov). Any discrepancies and disagreements were resolved by a third author (Torsten Doenst). Titles and abstracts were reviewed against pre-defined inclusion and exclusion criteria. Studies were considered for inclusion if they were written in English and reported direct comparison between patients with liver dysfunction and patients with no or mild liver dysfunction undergoing open heart surgery. The severity of liver dysfunction was evaluated with preoperative model for end-stage liver disease (MELD) score and/or Child–Turcotte–Pugh (CTP) score. Patients were stratified in the high score group (with liver dysfunction) and the low score group (no or mild liver dysfunction) based on the cutoffs proposed by each study as previously published [9–11]. Animal studies, abstracts, case reports, commentaries, editorials, expert opinions, conference presentations and studies not reporting the outcomes of interest were excluded. The full text was pulled for the selected studies for a second round of eligibility screening. References for articles selected were also reviewed for relevant studies not captured by the original search.

The quality of the included studies was assessed using the Newcastle-Ottawa Scale for observational studies (Supplementary Material, Table S2).

Two reviewers (Tulio Caldonazo and Hristo Kirov) independently performed data extraction, and the accuracy was verified by a third author (Torsten Doenst). The variables included were: study characteristics (publication year, country, sample size and type of surgery), patient demographics (age, sex, left ventricular ejection fraction, hypertension, diabetes mellitus, prior cerebrovascular accident, prior myocardial infarction and chronic renal failure) and postoperative characteristics [perioperative mortality, perioperative neurological events, prolonged ventilation, sepsis, bleeding and/or need for transfusion, acute kidney injury (AKI) and long-term mortality]. For studies that categorized patients using both the MELD and CTP score, all available data were used for the analysis.

### Outcomes

The primary outcome was perioperative mortality. The secondary outcomes were perioperative neurological events, prolonged ventilation, sepsis, bleeding and/or need for transfusion, AKI and long-term mortality. Perioperative outcomes were defined as in-hospital and 30-day events. Individual study definitions were used for all outcomes (Supplementary Material, Table S3). Subgroup analyses were performed for studies in which liver dysfunction was evaluated with the MELD score only and for studies in which it was evaluated with the CTP score only.

### Score definitions

Prognostic models are important tools for estimating disease severity and survival. Therefore, they serve as helpful medical decision-making tools for guiding patient care. These models were developed using statistical methodologies associated with the effects of variables of interest (e.g. demographics, clinical data and laboratory values). The MELD score was a prospectively developed and validated chronic liver disease severity scoring system that uses patient laboratory values (serum bilirubin, creatinine, sodium and the international normalized ratio for prothrombin time) to predict 3-month survival [12]. The CTP score involves the presence of ascites and hepatic encephalopathy and the serum albumin and international normalized ratio [13].

### Statistical analysis

Short-term binary outcomes were reported as odds ratios (ORs) with 95% confidence intervals (CIs) using the generic inverse variance method. For long-term mortality, incidence rate ratio (IRR) with its 95% CIs was reported using generic inverse variance method. IRR was estimated through several means based on the available study data. When hazard ratios were provided, the natural logarithm of the hazard ratio was used, otherwise IRR was estimated through the reported events and accumulated group-specific person-years of follow-up. Random effect meta-analysis was performed using ‘metafor’ and ‘meta’ packages. Publication bias was assessed by funnel plot (using the trim and fill method) and Egger test. Heterogeneity was reported as low (*I*^2^ = 0–25%), moderate (*I*^2^ = 26–50%) or high (*I*^2^ > 50%). Leave-one-out analyses for the primary outcome was performed to assess the robustness of the obtained estimate. *P*-value for interaction was used to ascertain subgroup differences.

All statistical analyses were performed using R (version 3.3.3, R Project for Statistical Computing) within RStudio.

## RESULTS

### Study and patient characteristics

A total of 7563 studies were retrieved from the systematic search, of which 33 met the criteria for inclusion in the final analysis. The PRISMA flowchart for study selection is provided in Supplementary Material, Fig. S1. Included studies were published between 1998 and 2020, all were observational cohort studies and 4 were multicentre. Eleven originated from the USA, 8 from Japan, 4 from Germany and the remaining from other countries. Sixteen studies used the CTP score to classify the severity of the liver dysfunction, 13 used the MELD score and 4 used both. Details of the included studies are provided in Table 1.

**Table 1:** Summary of included studies [references (superscript numbers) are reported in the Supplementary Material)

Study	Year	Country	Study period	Type of surgery	No patients with CTP or MELD score	No patients per arm
Part 1						
Liver dysfunction score used: CTP and MELD						
Garatti *et al.*^1^	2020	Italy	2000–2017	CABG, valve surgery	144	CTP A: 98 CTP B/C: 46 Low MELD: 118 High MELD: 26
Morimoto *et al.*^2^	2013	Japan	1999–2009	CABG, valve surgery, aortic surgery	32	CTP A: 14 CTP B/C: 18 Low MELD: 10 High MELD: 22
Thielmann *et al.*^3^	2010	Germany	1998–2008	CABG, valve surgery, other	57	CTP A: 39 CTP B/C: 18 Low MELD: 32 High MELD: 25
Vanhuyse *et al.*^4^	2012	France	1996–2010	CABG, valve surgery, aortic surgery, other	34	CTP A: 22 CTP B/C: 12 Low MELD: 27 High MELD: 7
Liver dysfunction score used: CTP only						
An *et al.*^5^	2007	China	1996–2005	CABG, valve surgery, aortic surgery, pericardiectomy, other	24	CTP A: 17 CTP B/C: 7
Arif *et al.*^6^	2012	Germany	2001–2011	CABG, valve surgery, aortic surgery, heart transplants, LVAD implantation, other	109	CTP A: 74 CTP B/C: 35
Bizouarn *et al.*^7^	1999	France	1995–1997	CABG, valve surgery, aortic surgery, heart transplants, LVAD implantation, other	12	CTP A: 10 CTP B/C: 2
Filsoufi *et al.*^8^	2007	USA	1998–2004	CABG, valve surgery, aortic surgery, pericardiectomy	27	CTP A: 10 CTP B/C: 17
Hayashida *et al.*^9^	2004	Japan	1989–2003	CABG, valve surgery, aortic surgery, pericardiectomy, other	18	CTP A: 10 CTP B/C: 8
Kaplan *et al.*^10^	2002	Turkey	1996–2000	CABG, valve surgery	10	CTP A: 6 CTP B/C: 4
Klemperer *et al.*^11^	1998	USA	1990–1996	CABG, valve surgery	13	CTP A: 8 CTP B/C: 5
Komoda *et al.*^12^	2013	Germany	1996–2011	Pericardiectomy	64	CTP A: 45 CTP B/C: 19
Lin *et al.*^13^	2014	Taiwan	1993–2012	CABG, valve surgery	55	CTP A: 30 CTP B/C: 25
Part 2						
Liver dysfunction score used: CTP only						
Lopez-Delgado *et al.*^14^	2012	Spain	2004–2009	CABG, valve surgery	58	CTP A: 34 CTP B/C: 24
Macaron *et al.*^15^	2012	USA	1992–2009	CABG, valve surgery	54	CTP A: 44 CTP B/C: 10
Morisaki *et al.*^16^	2010	Japan	1991–2009	CABG, valve surgery, aortic surgery	42	CTP A: 30 CTP B/C: 12
Murashita *et al.*^17^	2009	Japan	2002–2006	CABG, valve surgery	12	CTP A: 6 CTP B/C: 6
Sugimura *et al.*^18^	2012	Japan	2001–2010	CABG, valve surgery, aortic surgery, pericardiectomy, other	13	CTP A: 7 CTP B/C: 6
Suman *et al.*^19^	2004	USA	1992–2002	CABG, valve surgery, pericardiectomy	44	CTP A: 31 CTP B/C: 13
Yamane *et al.*^20^	2008	Japan	1999–2007	CABG, valve surgery, pericardiectomy, other	21	CTP A: 13 CTP B/C: 8
Liver dysfunction score used: MELD only						
Ailawadi *et al.*^21^	2009	USA	1994–2008	Valve surgery	168	Low MELD: 131 High MELD: 37
Chokshi *et al.*^22^	2012	USA	1998–2008	Heart transplant	617	Low MELD: 314 High MELD: 303
Deo *et al.*^23^	2013	USA	2007–2011	LVAD implantation	68	Low MELD: 49 High MELD: 19
Grimm *et al.*^24^	2015	USA	2000–2012	Heart transplant	22 597	Low MELD: 11174 High MELD: 11423
Hawkins *et al.*^25^	2019	USA	2011–2016	CABG, valve surgery, aortic surgery	21 272	Low MELD: 15566 High MELD: 5706
Part 3						
Liver dysfunction score used: MELD only						
Loforte *et al.*^26^	2019	Italy	2000–2018	Heart transplant	425	Low MELD: 100 High MELD: 325
Murata *et al.*^27^	2016	Japan	2008–2013	CABG, valve surgery, other	1856	Low MELD: 1449 High MELD: 407
Ortiz-Bautista *et al.*^28^	2018	Spain	2005–2015	Heart transplant	190	Low MELD: 78 High MELD: 112
Radakovic *et al.*^29^	2018	Germany	2009–2016	Pericardiectomy	79	Low MELD: 67 High MELD: 12
Tsuda *et al.*^30^	2013	Japan	1991–2011	CABG, valve surgery, pericardiectomy, other	168	Low MELD: 114 High MELD: 54
Woolley *et al.*^31^	2015	USA	2001–2011	LVAD implantation	63	Low MELD: 47 High MELD: 16
Yalcin *et al.*^32^	2020	Netherlands	2004–2017	LVAD implantation	290	Low MELD: 99 High MELD: 191
Yang *et al.*^33^	2012	USA	2000–2010	LVAD implantation	255	Low MELD: 176 High MELD: 79

CABG: coronary artery bypass grafting; CTP: Child–Turcotte–Pugh; MELD: model for end-stage liver disease; LVAD: left ventricular assist device.

A total of 48 891 patients were included in the final analysis, and the number of patients in each study ranged from 10 to 22 597. Twenty-nine thousand nine hundred twelve patients were included in the low score group and 18 979 in the high score group. Demographic data of the patient population in each study are summarized in Supplementary Material, Table S4. The mean age ranged from 47.0 to 70.1 years in the low score group and from 48.5 to 71.0 in the high score group. Percentage of male patients ranged from 25.0% to 87.5% in the low score group and from 33.3% to 88.0% in the high score group. The prevalence of hypertension ranged from 8.0% to 82.6% in the low score group and 16.0% to 89.0% in the high score group. The prevalence of diabetes mellitus ranged from 3.5% to 60.0% in the low score group and 8.0% to 50.5% in the high score group.

### Meta-analysis

#### Primary outcome

**Figure 1:**
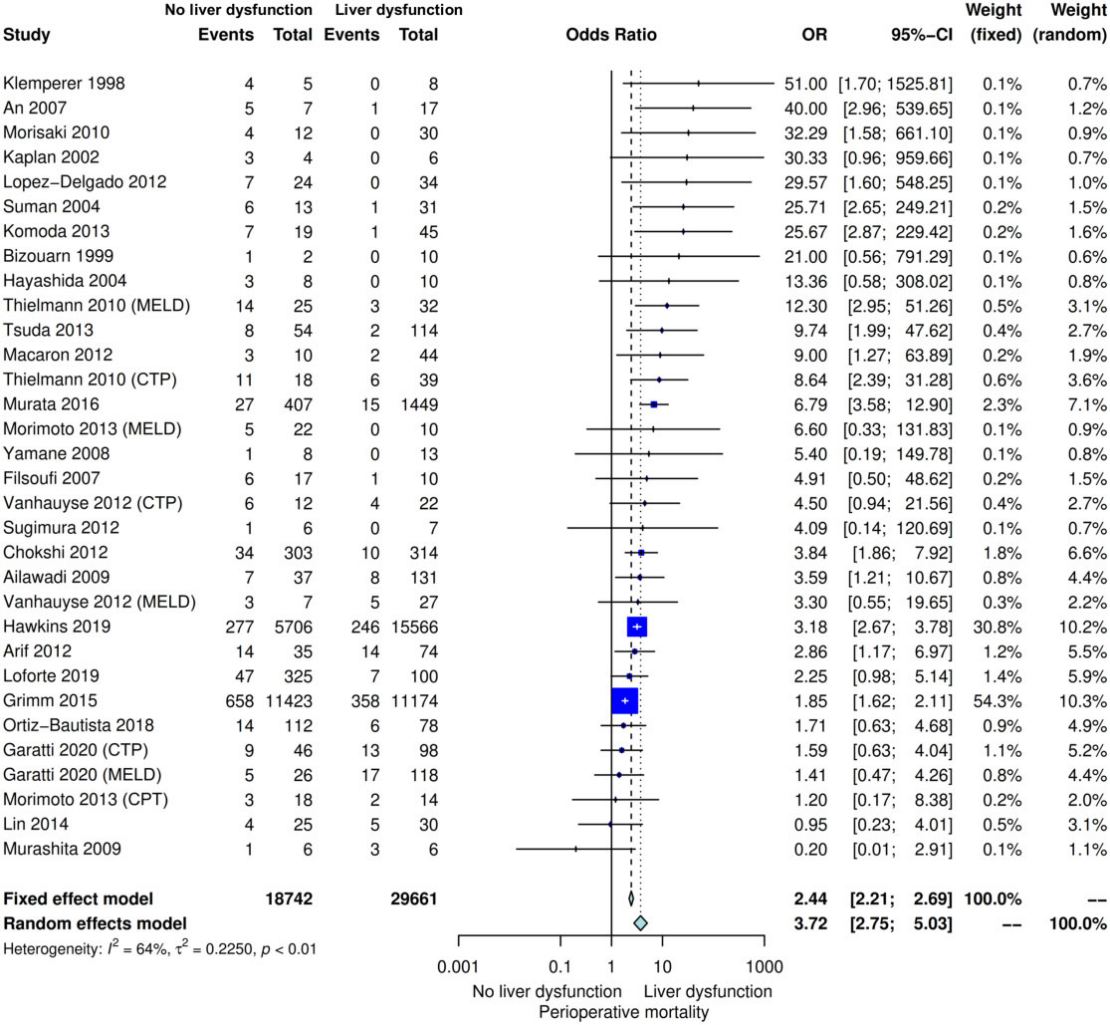
Forest plot for the primary outcome of perioperative mortality. CI: confidence interval; CTP: Child–Turcotte–Pugh; MELD: model of end-stage liver disease; OR: odds ratio.

Compared with low score, high score was associated with significantly higher perioperative mortality (OR 3.72, 95% CI 2.75–5.03, *P* < 0.001) (Fig. 1 and Table 2). Leave-one-out analysis confirmed the robustness of the main analysis (Supplementary Material, Fig. S2). Publication bias assessment is provided in Supplementary Fig. S3.

**Table 2:** Outcomes summary

Outcome	Number of studies	Measured estimate	Effect estimate (95% CI)	Higher in
Perioperative mortality (primary)	32	OR	3.72 [2.75; 5.03], *P* < 0.001	High score
CTP	20	OR	5.43 [2.96; 9.96]	High score
MELD	12	OR	3.18 [2.25; 4.50]	High score
Perioperative neurological events	12	OR	1.49 [1.30; 1.71], *P* < 0.001	High score
CTP	8	OR	1.49 [1.30; 1.70]	High score
MELD	4	OR	3.17 [0.66; 15.13]	High score
Prolonged ventilation	15	OR	2.45 [1.94; 3.09], *P* < 0.001	High score
CTP	8	OR	3.31 [1.54; 7.11]	High score
MELD	7	OR	2.38 [1.79; 3.16]	High score
Sepsis	10	OR	3.88 [2.07; 7.26], *P* < 0.001	High score
CTP	4	OR	4.01 [1.76; 9.13]	High score
MELD	6	OR	3.70 [1.40; 9.76]	High score
Bleeding and/or need for transfusion	16	OR	1.95 [1.43; 2.64], *P* < 0.001	High score
CTP	5	OR	2.12 [1.45; 3.10]	High score
MELD	11	OR	1.84 [1.02; 3.34]	High score
Acute Kidney Injury	17	OR	3.84 [2.12; 6.98], *P* < 0.001	High score
CTP	9	OR	3.18 [1.61; 6.28]	High score
MELD	8	OR	4.08 [1.87; 8.87]	High score
Long-term mortality	16	IRR	1.29 [1.14; 1.46], *P* < 0.001	High score
CTP	8	IRR	1.74 [1.20; 2.53]	High score
MELD	8	IRR	1.21 [1.07; 1.37]	High score

CI: confidence interval; CTP: Child–Turcotte–Pugh; IRR: incidence rate ratio; MELD: model for end-stage liver disease; OR: odds ratios.

#### Subgroup analysis of the primary outcome

**Figure 2:**
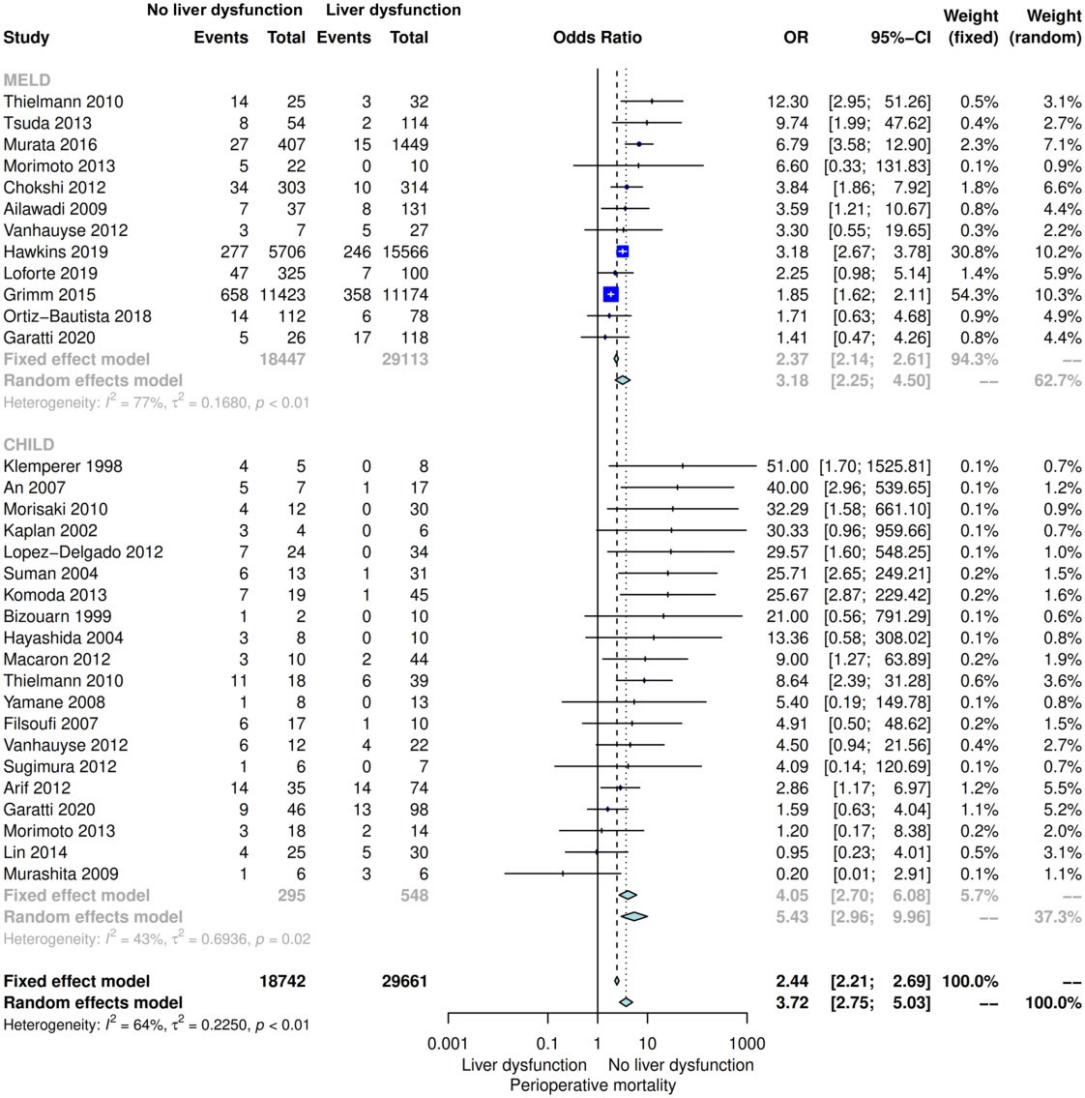
Subgroup analysis for perioperative mortality for studies using the MELD score versus the CTP score. CI: confidence interval; CTP: Child–Turcotte–Pugh; MELD: model of end-stage liver disease; OR: odds ratio.

The results of the subgroup analyses were consistent with the primary analysis. The OR for perioperative mortality was 3.18 (95% CI 2.25–4.50) for studies in which the MELD score was used and 5.43 (95% CI 2.96–9.96) for studies in with the CTP score was used (*P* for interaction: 0.13) (Fig. 2).

#### Secondary outcomes

Compared with low score, high score was associated with a significantly higher rate of perioperative neurological events (OR 1.49, 95% CI 1.30–1.71, *P* < 0.001), prolonged ventilation (OR 2.45, 95% CI 1.94–3.09, *P* < 0.001), sepsis (OR 3.88, 95% CI 2.07–7.26, *P* < 0.001), bleeding and/or need for transfusion (OR 1.95, 95% CI 1.43–2.64, *P* < 0.001), AKI (OR 3.84, 95% CI 2.12–6.98, *P* < 0.001) and long-term mortality (IRR 1.29, 95% CI 1.14–1.46, *P* < 0.001) (Table 2).

## DISCUSSION

Our analysis suggests that liver dysfunction in patients after cardiac surgery is associated with higher short- and long-term mortality and increased occurrence of most perioperative adverse events. Specifically, the high MELD or CTP score was associated with significantly increased perioperative mortality, higher rate of perioperative neurological events, prolonged ventilation, sepsis, bleeding and/or need for transfusion and AKI.

Although the findings of liver dysfunction being associated with worse outcome may not be surprising, or relevant because it appears to affect only a small number of patients. While this impression may be true for patients with preoperatively diagnosed liver cirrhosis, it is not for all patients with liver dysfunction *per se*. Patients with liver cirrhosis reflect <1% of all patients in the STS database [14]. However, if one assumes that the liver does not function normally anymore at a MELD score above 9, 27% of all patients in the STS database would be affected [7]. Thus, this recent database analysis demonstrates that the number of patients with evidence for liver dysfunction in the cardiac surgical population is much higher than currently expected. In fact, it is at least comparable to the number of patients developing new-onset postoperative atrial fibrillation. Several publications out of the STS database have shown an incidence of postoperative atrial fibrillation of 19% (for all cardiac surgeries) [15] or 29% for isolated coronary bypass surgery [16]. Interestingly, the literature reveals only half as many publications addressing liver dysfunction than postoperative atrial fibrillation having analysed only one-fifth of patients [17] compared to postoperative atrial fibrillation studies.

It is therefore highly important to systematically review the negative effects of liver dysfunction on classic short and long-term cardiovascular adverse events. This meta-analysis provides important information for clinical decision-making and patient information. Appropriately assessing preoperative risk is most important for the patient, specifically if the requirement for cardiac surgery is free of alternatives (e.g. infective endocarditis). For instance, we demonstrated in a cohort of patients requiring surgery for infective endocarditis that preoperative liver dysfunction was a tremendous risk factor for death. However, we also showed that those patients who survived the hospital stay, had similar 5-year survival and quality of life to those patients not affected by pre- or perioperative liver dysfunction [18].

In such cases where surgery is without alternatives, being aware of the increased risk (reflected by increased MELD scores for instance) may help with defining a suitable operative strategy. Gopaldas *et al.* [14] identified in over 3 million patients from the STS database that the use of cardiopulmonary bypass is an independent risk factor for mortality in patients with liver cirrhosis undergoing coronary artery bypass grafting. Use of cardiopulmonary bypass increased the odds for mortality 4.6-fold [14]. When performed off-pump, the presence of cirrhosis did not affect mortality or morbidity unless there was severe liver dysfunction [14]. Therefore, the avoidance of cardiopulmonary bypass and cardioplegia at least in isolated coronary cases might be a successful strategy in patients with liver dysfunction. However, the evidence in support of this conclusion by Gopaldas *et al.* does not exist, yet.

Although there seems to be a clear association between liver dysfunction and adverse events after cardiac surgery, some of the most commonly used risk evaluation models do not take it into consideration [1], and other seem not to adequately assess the risks [7]. The results of our analysis provide a previously unknown quantification of dimensions of these relationships. The association of liver dysfunction not only with mortality, but also with neurological events, prolonged ventilation, sepsis, bleeding and/or need for transfusion and AKI may help in preoperative patient counselling, and even estimating individually varying financial investments. Furthermore, the results of our work suggest that routine evaluation of liver scores might be helpful for preoperative evaluation of patients in cardiac surgery and may assist identifying high-risk patients in cases when classic surgical scores fail. Therefore, we believe that this work will contribute to raising the awareness of the importance of properly assessing for liver dysfunction in patients undergoing open cardiac surgery.

To the best of our knowledge, this comprehensive meta-analysis is the first to include studies with both CTP and MELD scores and provide a broad overview of various clinical outcomes and their association with liver dysfunction after cardiac surgery. The findings are relevant because an increasing number of patients undergoing cardiac surgery have liver dysfunction [6].

### Limitations

This work has the intrinsic limitations of trial-level meta-analyses of observational series, including the risk of methodological heterogeneity of the included studies. Studies have included different numbers of patients with 2 studies including the majority of them. Furthermore, patient management among included studies was performed according to individual centre strategy or protocol, with heterogeneous approaches and diverse indications, which may limit data pooling or the definitive value of the conclusions.

## CONCLUSION

This analysis suggests that liver dysfunction in patients undergoing cardiac surgery is associated with the higher risk of short- and long-term mortality and also with an increased occurrence of various perioperative adverse events.

## Supplementary Material

ivac280_Supplementary_Data

## Data Availability

The data underlying this article are available in the article and in its online supplementary material.

## ABBREVIATIONS

AKI: Acute kidney injury
CI: Confidence interval
CTP: Child–Turcotte–Pugh
IRR: Incidence rate ratio
MELD: Model of end-stage liver disease
OR: Odds ratio
