# Supplementary material for: Association of liver dysfunction with outcomes after cardiac surgery—a meta-analysis
Source: Interact Cardiovasc Thorac Surg. 2022 Dec 8;35(6):ivac280. doi: 10.1093/icvts/ivac280 (PMC9741516; doi:10.1093/icvts/ivac280)
Supplement: ivac280_Supplementary_Data [file ivac280_supplementary_data.docx]

**Supplementary Appendix**

**Supplementary Table 1**: Search strategy for Ovid MEDLINE.

**Supplementary Table 2**: Assessment of risk of bias using the Newcastle Ottawa Scale.

**Supplementary Table 3**: Definition of the outcomes of interest by study.

**Supplementary Table 4**: Demographics of included patients.

**Supplementary Table 5:** The MELD Score value of each individual group from all the studies.

**Supplementary Figure 1**: Preferred Reporting Items for Systematic Reviews and Meta-Analyses (PRISMA) flow diagram.

**Supplementary Figure 2**: Leave-one-out analysis for perioperative mortality.

**Supplementary Figure 3**: Funnel plot for assessment of publication bias for the primary outcome of perioperative mortality.

**Supplementary references**

**Supplementary Table 1:** Search strategy for Ovid MEDLINE.

| 1 Cardiac Surgical Procedures/ or (cardiac surgery or cardiac surgical procedure* or heart surgery or heart valve surgery or heart surgical procedures* or cardiac operation* or heart operation* or cardiosurgery or myocardial resection).tw. |
| --- |
| 2 Mitral Valve Annuloplasty/ or ((bicuspid cardiac valve or bicuspid cardiac valvular or bicuspid heart valve or bicuspid heart valvular or bicuspid or bicuspid valve or bicuspid valvular or left atrioventricular cardiac valve or left atrioventricular heart valve or left atrioventricular valvular or mitral cardiac valve or mitral cardiac valvular or mitral heart valve or mitral heart valvular or mitral or mitral valvular) adj2 (annuloplast* or repair or replacement)).tw. |
| 3 Atherectomy, Coronary/ or (coronary atherectom* or rotational atherectom*).tw |
| 4 Coronary Artery Bypass/ or Coronary Artery Bypass, Off-Pump/ or (CABG or aorticocoronary anastomosis or total arterial revasculari*ation* or multiple arterial revasculari*ation*).tw. |
| 5 (coronary adj2 (bypass* or graft* or surger*)).tw. |
| 6 Internal Mammary-Coronary Artery Anastomosis/ or ((right internal mammary artery or RIMA or left internal mammary artery or LIMA or Coronary Internal Mammary Artery or arteria mammaria interna or arteria thoracica interna or internal thoracic artery or mammary internal artery) and (transplant* or graft* or anastomosis)).tw. |
| 7 (surgical revasculari*ation* or cardiac muscle revasculari*ation* or coronary revasculari*ation* or heart muscle revasculari*ation* or heart myocardium revasculari*ation* or heart revasculari*ation* or internal mammary arterial anastomosis or internal mammary arterial implant* or internal mammary artery anastomosis or internal mammary artery graft* or internal mammary artery implant* or internal mammary-coronary artery anastomosis).tw. |
| 8 Myocardial Revascularization/ or (myocardial revasculari*ation* or myocardium revasculari*ation* or mammary artery implant* or mammary arterial implant* or mammary artery reimplant* or mammary arterial reimplant* or vineberg operation*).tw. |
| 9 Pericardiectomy/ or (pericardiectomy or pericardiectomies or pericardectomy or pericardectomies or pericardiotomy or pericardiotomies or pericardotomy or pericardotomies).tw. |
| 10 Heart Valve Prosthesis Implantation/ or (heart valve prosthesis implantation or heart valve prosthesis implant).tw. |
| 11 Cardiac Valve Annuloplasty/ or (Cardiac Valve Annuloplasty or Cardiac Valve Annuloplasties or Valvular Annuloplasties or Valvular Annuloplasty or Heart Valve Annuloplasty or Heart Valve Annuloplasties or Cardiac Valve Annulus Repair or Heart Valve Annulus Repair or Cardiac Valve Annular Repair or Heart Valve Annular Repair or Cardiac Valve Annular Reduction or Cardiac Valve Annulus Shortening or Cardiac Valve Annulus Reduction).tw. |
| 12 Heart Transplantation/ or (heart transplantation* or heart transplant or heart grafting* or cardiac transplantation* or cardiac transplant).tw. |
| 13 Heart Bypass, Right/ or (right heart bypass or right heart bypasses or cavopulmonary anastomosis or cavopulmonary anastomoses or cavopulmonary shunt or cavopulmonary shunts).tw. |
| 14 Heart-Lung Transplantation/ or (heart lung transplant* or heart lung graft*).tw |
| 15 Circulatory Arrest, Deep Hypothermia Induced/ or (hypothermic circulatory arrest or hypothermic cardiac arrest or hypothermic circulation arrest or hypothermia induced circulatory arrest or hypothermia induced cardiac arrest or hypothermia induced circulation arrest or hypothermic arrest or DHCA).tw |
| 16 or/1-15 |
| 17 Liver Diseases/ or (liver disease* or liver dysfunction* or liver disorder* or hepatic disease* or hepatic disorder* or hepatic dysfunction* or hepatopathy or liver cell disease* or liver illness* or liver injur*) |
| 18 alpha 1-Antitrypsin Deficiency/ or (alpha 1-Antitrypsin Deficienc* or alpha 1 proteinase inhibitor deficienc* or antitrypsin alpha 1 deficienc* or mckusick 10740).tw |
| 19 Chemical and Drug Induced Liver Injury/ or "Chemical and Drug Induced Liver Injury, Chronic"/ or (liver toxic* or toxic liver).tw |
| 20 Liver Cirrhosis, Biliary/ or (billary cirrhosis or billary cholangitides).tw |
| 21 Fatty Liver/ or Fatty Liver, Alcoholic/ or Non-alcoholic Fatty Liver Disease/ or (fatty liver or steatohepatitis* or "steatosis of the liver" or "steatosis of liver" or visceral steatos* or liver steatos* or NAFLD).tw |
| 22 Hepatic Insufficiency/ or (hepatic insufficienc* or liver insufficienc*).tw |
| 23 Liver Failure/ or End Stage Liver Disease/ or (liver failure or hepatic failure).tw |
| 24 Hepatic Encephalopathy/ or (hepatic encephalopath* or portal systemic encephalopath* or hepatocerebral encephalopath* or portosystemic encephalopath* or hepatic coma* or hepatic stupor*).tw |
| 25 exp Hepatitis/ or (hepatitis or hepatitides).tw |
| 26 exp Liver Cirrhosis/ or (liver cirrhosis or "cirrhosis of the liver" or hepatic cirrhosis or liver fibrosis).tw |
| 27 Liver Diseases, Alcoholic/ or Fatty Liver, Alcoholic/ or Hepatitis, Alcoholic/ or Liver Cirrhosis, Alcoholic/ |
| 28 or/17-27 |
| 29 16 and 28 |
| 30 limit 29 to English language |

**Ovid MEDLINE** (ALL – 1946 to present)

Searched on Feb 24, 2021

Limited to English language

No article type or publication date restriction

**Supplementary Table 2:** Assessment of risk of bias using the Newcastle Ottawa Scale.

| STUDY | SELECTION | COMPARABILITY | OUTCOME/EXPOSURE |
| --- | --- | --- | --- |
| Garatti, 2020^1^ | **** | ** | *** |
| Morimoto, 2013^2^ | **** | * | *** |
| Thielmann, 2010^3^ | **** |  | *** |
| Vanhuyse, 2012^4^ | **** |  | *** |
| An, 2007^5^ | **** |  | ** |
| Arif, 2012^6^ | **** |  | *** |
| Bizouarn, 1999^7^ | **** |  | *** |
| Filsoufi, 2007^8^ | **** |  | *** |
| Hayashida, 2004^9^ | **** |  | ** |
| Kaplan, 2002^10^ | **** |  | *** |
| Klemperer, 1998^11^ | **** |  | *** |
| Komoda, 2013^12^ | **** | * | *** |
| Lin, 2014^13^ | **** | * | *** |
| Lopez-Delgado, 2012^14^ | **** |  | *** |
| Macaron, 2012^15^ | **** |  | *** |
| Morisaki, 2010^16^ | **** |  | *** |
| Murashita, 2009^17^ | **** |  | ** |
| Sugimura, 2012^18^ | **** |  | *** |
| Suman, 2004^19^ | **** |  | *** |
| Yamane, 2008^20^ | **** |  | *** |
| Ailawadi, 2009^21^ | **** | * | *** |
| Chokshi, 2012^22^ | *** | * | *** |
| Deo, 2013^23^ | **** |  | *** |
| Grimm, 2015^24^ | *** | * | *** |
| Hawkins, 2019^25^ | **** | * | *** |
| Loforte. 2019^26^ | *** | ** | *** |
| Murata, 2016^27^ | **** | * | *** |
| Ortiz-Bautista, 2018^28^ | *** | * | *** |
| Radakovic, 2018^29^ | **** |  | *** |
| Tsuda, 2013^30^ | **** | ** | *** |
| Wooley, 2015^31^ | *** | * | *** |
| Yalcin, 2020^32^ | *** | ** | *** |
| Yang, 2012^33^ | *** | ** | *** |

**Supplementary Table 3**: Definition of the outcomes of interest by study.

| STUDY | NEUROLOGICAL EVENT | ACUTE KIDNEY INJURY | PROLONGED VENTILATION | SEPSIS | TRANSFUSION/ BLEEDING |
| --- | --- | --- | --- | --- | --- |
| Garatti, 2020^1^ | Stroke | Acute kidney injury and continuous veno-venous hemodialysis | Pulmonary dysfunction | Sepsis | Transfusion |
| Morimoto, 2013^2^ | Stroke | Renal failure | Prolonged ventilation | Sepsis | Bleeding |
| Vanhuyse, 2012^4^ | Not reported | Not reported | Not reported | Not reported | Patients receiving transfusion |
| Bizouarn, 1999^7^ | Not reported | Not reported | Not reported | Multiple organ failure | Not reported |
| Filsoufi, 2007^8^ | Not reported | Renal failure requiring dialysis | Prolonged ventilation/respiratory failure | Sepsis | Reoperation for bleeding |
| Hayashida, 2004^9^ | Not reported | Renal failure | Respiratory failure | Not reported | Bleeding |
| Kaplan, 2002^10^ | Not reported | Not reported | Extended intubation | Sepsis | Revision due to hemorrhage or gastrointestinal system bleeding |
| Klemperer, 1998^11^ | Stroke | Renal complication | Pulmonary complication | Not reported | Re-exploration for mediastinal bleeding |
| Lin, 2014^13^ | Not reported | Not reported | Not reported | Not reported | Bleeding |
| Macaron, 2012^15^ | Not reported | Renal failure | > 24 hours | Not reported | Reoperation for bleed/tamponade |
| Murashita, 2009^17^ | Intracranial hemorrhage | Renal failure | Not reported | Multiple organ failure | Gastrointestinal bleeding or re-exploration for bleeding |
| Sugimura, 2012^18^ | Cerebral infarction | Dialysis | Not reported | Not reported | Not reported |
| Yamane, 2008^20^ | Not reported | New dialysis | > 48 hours | Not reported | Not reported |
| Ailawadi, 2009^21^ | Stroke | Hemodialysis | Prolonged ventilation | Sepsis | Not reported |
| Chokshi, 2012^22^ | Stroke | Renal insufficiency | Respiratory failure | Not reported | Re-operation for bleeding |
| Grimm, 2015^24^ | Stroke | Not reported | Not reported | Not reported | Not reported |
| Hawkins, 2019^25^ | Permanent stroke | Dialysis-dependent renal failure | Prolonged ventilation | Not reported | Not reported |
| Loforte. 2019^26^ | Not reported | Continuous veno-venous hemofiltration | > 48 hours | Not reported | Re-thoracotomy for bleeding |
| Murata, 2016^27^ | Stroke | Continuous hemofiltration/hemodialysis | Not reported | Not reported | Not reported |
| Tsuda, 2013^30^ | Stroke | Hemodialysis | > 2 days | Sepsis | Not reported |
| Wooley, 2015^31^ | Not reported | Not reported | Not reported | Not reported | Bleeding |
| Yalcin, 2020^32^ | Ischemic or hemorrhagic event | Not reported | Not reported | Not reported | Not reported |

**Supplementary Table 4:** Demographics of included patients (part 1).

| Study | Age (mean±SD) | | Male (%) | | Mean LVEF (%) | | HPT (%) | | DM (%) | | Prior CVA (%) | | Prior MI (%) | | Chronic renal failure (%) | |
| --- | --- | --- | --- | --- | --- | --- | --- | --- | --- | --- | --- | --- | --- | --- | --- | --- |
|  | **Low score** | **High score** | **Low score** | **High score** | **Low score** | **High score** | **Low score** | **High score** | **Low score** | **High score** | **Low score** | **High score** | **Low score** | **High score** | **Low score** | **High score** |
| Garatti, 2020 (CTP)^1^ | 66.0±9.0 | NR | 75.0 | 56.2 | 56.0 | NR | 58.0 | 67.4 | 44.0 | 43.5 | 10.0 | 13.0 | NR | NR | 51.0 | 63.0 |
| Garatti, 2020 (MELD)^1^ | 66.3±9.9 | 65.0±11.0 | 71.2 | 61.0 | 56.2 | 55.0 | 61.0 | 61.0 | 43.2 | 46.0 | 8.5 | 23.0 | NR | NR | 49.0 | 81.0 |
| Morimoto, 2013 (CTP)^2^ | 69.0±11.0 | NR | 57.0 | 72.3 | 51.0 | 57.0 | NR | NR | 50.0 | 50.0 | 29.0 | 16.6 | NR | NR | NR | NR |
| Morimoto, 2013 (MELD)^2^ | 68.0±13.0 | 71.0±8.0 | 50.0 | 72.7 | 61.0±8.0 | NR | NR | NR | 60.0 | 45.5 | 20.0 | 22.7 | NR | NR | 20.0 | 63.0 |
| Thielmann, 2010 (CTP)^3^ | NR | NR | NR | NR | NR | NR | NR | NR | NR | NR | NR | NR | NR | NR | NR | NR |
| Thielmann, 2010 (MELD)^3^ | NR | NR | NR | NR | NR | NR | NR | NR | NR | NR | NR | NR | NR | NR | NR | NR |
| Vanhuyse, 2012 (CTP)^4^ | NR | NR | NR | NR | NR | NR | NR | NR | NR | NR | NR | NR | NR | NR | NR | NR |
| Vanhuyse, 2012 (MELD)^4^ | NR | NR | NR | NR | NR | NR | NR | NR | NR | NR | NR | NR | NR | NR | NR | NR |
| An, 2007^5^ | NR | NR | NR | NR | NR | NR | NR | NR | NR | NR | NR | NR | NR | NR | NR | NR |
| Arif, 2012^6^ | NR | NR | NR | NR | NR | NR | NR | NR | NR | NR | NR | NR | NR | NR | NR | NR |
| Bizouarn, 1999^7^ | 60.5±8.6 | 48.5±23.5 | 70.0 | 50.0 | NR | NR | 30.0 | 50.0 | 20.0 | 0.0 | NR | NR | NR | NR | NR | NR |
| Filsoufi, 2007^8^ | NR | NR | NR | NR | NR | NR | NR | NR | NR | NR | NR | NR | NR | NR | NR | NR |
| Hayashida, 2004^9^ | 62.8±10.3 | 65.8±12.8 | 50.0 | 75.0 | NR | NR | 10.0 | 0.0 | 30.0 | 12.5 | NR | NR | NR | NR | 20.0 | 0.0 |
| Kaplan, 2002^10^ | 55±3.9 | 58.5±7.4 | 25.0 | 83.0 | NR | NR | NR | NR | NR | NR | NR | NR | NR | NR | NR | NR |
| Klemperer, 1998^11^ | 66.9±7.6 | 63±8.3 | 87.5 | 80.0 | NR | NR | NR | NR | 50.0 | 40.0 | 12.5 | 0.0 | NR | NR | 20.0 | 37.5 |
| Komoda, 2013^12^ | 52.4±15.0 | 62.9±9.7 | 68.9 | 84.2 | 55.8 | 52.8 | NR | NR | 15.6 | 26.3 | NR | NR | NR | NR | 2.2 | 10.5 |
| Lin, 2014^13^ | 64.5±3.8 | 53±4.5 | 73.0 | 84.0 | NR | NR | 40.0 | 16.0 | 43.0 | 8.0 | NR | NR | NR | NR | NR | NR |
| Lopez-Delgado, 2012^14^ | NR | NR | NR | NR | NR | NR | NR | NR | NR | NR | NR | NR | NR | NR | NR | NR |
| Macaron, 2012^15^ | 64.8±10.3 | 59.7±17.2 | 61.4 | 50.0 | NR | NR | NR | NR | NR | NR | NR | NR | NR | NR | NR | NR |
| Morisaki, 2010^16^ | NR | NR | NR | NR | NR | NR | NR | NR | NR | NR | NR | NR | NR | NR | NR | NR |
| Murashita, 2009^17^ | 70.1±9.4 | 69.6±8.5 | 50.0 | 33.3 | NR | NR | NR | NR | NR | NR | NR | NR | NR | NR | NR | NR |
| Sugimura, 2012^18^ | 61.8±11.5 | 60.1±11.8 | 85.7 | 66.6 | NR | NR | NR | NR | NR | NR | NR | NR | NR | NR | NR | NR |
| Suman, 2004^19^ | NR | NR | NR | NR | NR | NR | NR | NR | NR | NR | NR | NR | NR | NR | NR | NR |
| Yamane, 2008^20^ | NR | NR | NR | NR | NR | NR | NR | NR | NR | NR | NR | NR | NR | NR | NR | NR |
| Ailawadi, 2009^21^ | 67.0±4.5 | 55.0±5.5 | 35.1 | 70.3 | 55.0 | 55.0 | NR | NR | 20.6 | 21.6 | 16.0 | 16.2 | 19.1 | 16.2 | 13.0 | 51.4 |
| Chokshi, 2012^22^ | 50.1±12.4 | NR | 71.0 | 79.8 | 18.8 | NR | 36.0 | 37.2 | 27.0 | 27.0 | 14.0 | 9.2 | NR | NR | 9.0 | 15.8 |
| Deo, 2013^23^ | NR | NR | NR | NR | NR | NR | NR | NR | NR | NR | NR | NR | NR | NR | NR | NR |

CVA: cerebrovascular accident, DM: diabetes mellitus, HPT: hypertension, LVEF: left ventricular ejection fraction, MELD: model for end-stage liver disease, MI: myocardial infarction, NR: not reported, SD: standard deviation.

| Study | Age (mean±SD) | | Male (%) | | Mean LVEF (%) | | HPT (%) | | DM (%) | | Prior CVA (%) | | Prior MI (%) | | Chronic renal failure (%) | |
| --- | --- | --- | --- | --- | --- | --- | --- | --- | --- | --- | --- | --- | --- | --- | --- | --- |
|  | **Low score** | **High score** | **Low score** | **High score** | **Low score** | **High score** | **Low score** | **High score** | **Low score** | **High score** | **Low score** | **High score** | **Low score** | **High score** | **Low score** | **High score** |
| Grimm, 2015^24^ | NR | NR | 69.0 | 81.6 | NR | NR | NR | NR | 22.7 | 25.0 | NR | NR | NR | NR | NR | NR |
| Hawkins, 2019^25^ | 65±3.8 | 68.2 | 68.5 | 77.7 | 55.0 | 54.0 | 82.6 | 89.0 | 40.2 | 50.5 | 7.3 | 11.5 | NR | NR | 0.0 | 10.4 |
| Loforte. 2019^26^ | 49.4±11.7 | 53.5±11.4 | 66.0 | 84.6 | NR | NR | 8.0 | 18.5 | 13.0 | 24.3 | NR | NR | NR | NR | NR | NR |
| Murata, 2016^27^ | 66.1±12.6 | 68.9±10 | 68.6 | 71.7 | 59.7 | 52.4 | NR | NR | NR | NR | NR | NR | NR | NR | NR | NR |
| Ortiz-Bautista, 2018^28^ | 47.0±14.0 | 50.0±12.0 | 72.0 | 81.0 | NR | NR | 23.0 | 33.0 | 18.0 | 19.0 | NR | NR | NR | NR | NR | NR |
| Radakovic, 2018^29^ | NR | NR | NR | NR | NR | NR | NR | NR | NR | NR | NR | NR | NR | NR | NR | NR |
| Tsuda, 2013^30^ | 63.8±10.0 | 63.9±10.2 | 33.3 | 50.0 | 55.8 | 55.2 | NR | NR | 3.5 | 14.8 | 17.5 | 13.0 | NR | NR | 0.9 | 18.5 |
| Wooley, 2015^31^ | 51.0±16.0 | 57.0±14.0 | 66.0 | 88.0 | 15.0 | 15.0 | NR | NR | 32.0 | 38.0 | NR | NR | NR | NR | NR | NR |
| Yalcin, 2020^32^ | 54.0±19.0 | 56.0±19.0 | 64.0 | 82.0 | NR | NR | 49.0 | 44.0 | NR | NR | 12.0 | 21.0 | NR | NR | NR | NR |
| Yang, 2012^33^ | 53.1±14.8 | 57.3±11.2 | 79.0 | 84.8 | 17.6 | 18.6 | NR | NR | 33.3 | 38.9 | NR | NR | NR | NR | NR | NR |

**Supplementary Table 4:** Demographics of included patients (part 2).

CVA: cerebrovascular accident, DM: diabetes mellitus, HPT: hypertension, LVEF: left ventricular ejection fraction, MELD: model for end-stage liver disease, MI: myocardial infarction, NR: not reported, SD: standard deviation.

**Supplementary Table 5:** The MELD Score value of each individual group from all the studies.

| **STUDY** | **LOW SCORE** | **HIGH SCORE** |
| --- | --- | --- |
| Ailawadi, 2009 | MELD<15 | MELD≥15 |
| Thielmann, 2010 | MELD<13.5 | MELD≥13.5 |
| Yang, 2012 | MELD<17 | MELD≥17 |
| Chokshi, 2012 | MELD<14 | MELD≥14 |
| Vanhuyse, 2012 | MELD<15 | MELD≥15 |
| Deo, 2013 | MELD<19 | MELD≥19 |
| Morimoto, 2013 | MELD<10 | MELD≥10 |
| Tsuda, 2013 | MELD<7 | MELD≥7 |
| Woolley, 2015 | MELD<18 | MELD≥18 |
| Grimm, 2015 | MELD<12.6 | MELD≥12.6 |
| Murata, 2016 | MELD<12 | MELD≥12 |
| Ortiz-Bautista, 2018 | MELD-XI<12 | MELD-XI≥12 |
| Radakovic, 2018 | MELD<15.5 | MELD≥15.5 |
| Hawkins, 2019 | MELD<9 | MELD≥9 |
| Loforte, 2019 | MELD-XI<11 | MELD-XI≥11 |
| Garrati, 2020 | MELD<12 | MELD>12 |
| Yalcin, 2020 | MELD<12.6 | MELD≥12.6 |

**Supplementary Figure 1:** Preferred Reporting Items for Systematic Reviews and Meta-Analyses (PRISMA) flow diagram.

**Supplementary Figure 2:** Leave-one-out analysis for perioperative mortality.


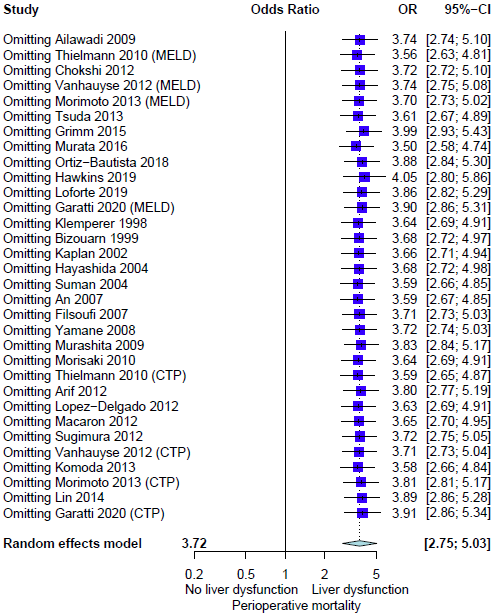


CI: confidence interval, OR: odds ratio.

**Supplementary Figure 3:** Funnel plot for assessment of publication bias for the primary outcome of perioperative mortality.


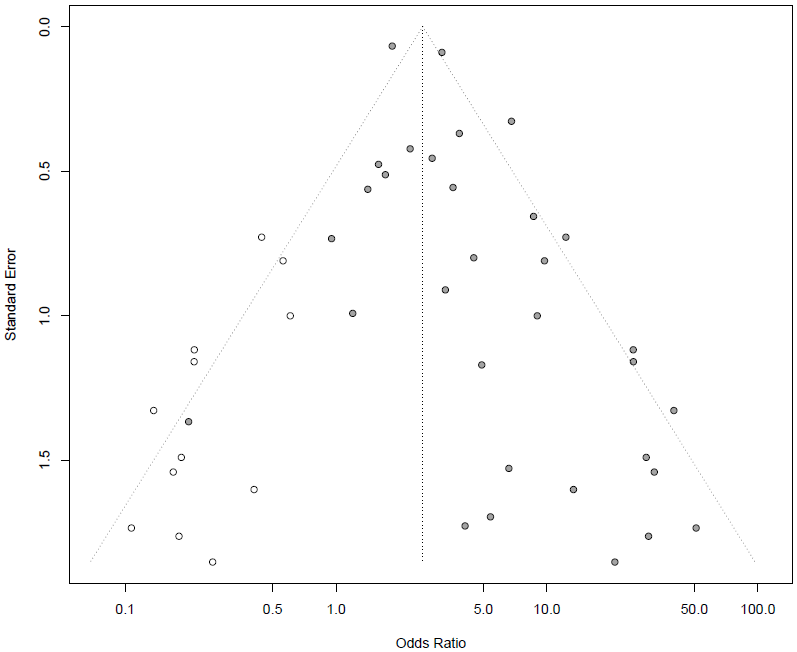


Funnel plot for assessment of publication bias for the primary outcome of perioperative mortality. Egger’s test [bias=1.0364±0.3085, P=0.0021) which reflects presence of publication bias, hence we did trim and fill method and the obtained bias-adjusted odds ratio was 2.5765 [1.9052; 3.4843], P< 0.0001 including 44 studies (with 12 added studies (white circles) from the trim and fill method to adjust for funnel plot asymmetry). The grey dots represent individual studies and the white dots represent potential bias source.

**Supplementary References**

1. Garatti A, Daprati A, Cottini M, Russo CF, Dalla Tomba M, Troise G, et al. Cardiac Surgery in Patients With Liver Cirrhosis (CASTER) Study: Early and Long-Term Outcomes. Ann Thorac Surg. 2021 Apr 1;111(4):1242–51.

2. Morimoto N, Okada K, Okita Y. The Model for End-Stage Liver Disease (MELD) Predicts Early and Late Outcomes of Cardiovascular Operations in Patients With Liver Cirrhosis. Ann Thorac Surg. 2013 Nov 1;96(5):1672–8.

3. Thielmann M, Mechmet A, Neuhäuser M, Wendt D, Tossios P, Canbay A, et al. Risk prediction and outcomes in patients with liver cirrhosis undergoing open-heart surgery☆. Eur J Cardiothorac Surg. 2010 Nov 1;38(5):592–9.

4. Vanhuyse F, Maureira P, Portocarrero E, Laurent N, Lekehal M, Carteaux J-P, et al. Cardiac surgery in cirrhotic patients: results and evaluation of risk factors. Eur J Cardiothorac Surg. 2012 Aug 1;42(2):293–9.

5. An Y, Xiao YB, Zhong QJ. Open-Heart Surgery in Patients with Liver Cirrhosis: Indications, Risk Factors, and Clinical Outcomes. Eur Surg Res. 2007;39(2):67–74.

6. Arif R, Seppelt P, Schwill S, Kojic D, Ghodsizad A, Ruhparwar A, et al. Predictive Risk Factors for Patients With Cirrhosis Undergoing Heart Surgery. Ann Thorac Surg. 2012 Dec 1;94(6):1947–52.

7. Bizouarn P, Ausseur A, Desseigne P, Le Teurnier Y, Nougarede B, Train M, et al. Early and late outcome after elective cardiac surgery in patients with cirrhosis. Ann Thorac Surg. 1999 May;67(5):1334–8.

8. Filsoufi F, Salzberg SP, Rahmanian PB, Schiano TD, Elsiesy H, Squire A, et al. Early and late outcome of cardiac surgery in patients with liver cirrhosis. Liver Transpl. 2007;13(7):990–5.

9. Hayashida N, Shoujima T, Teshima H, Yokokura Y, Takagi K, Tomoeda H, et al. Clinical outcome after cardiac operations in patients with cirrhosis. Ann Thorac Surg. 2004 Feb 1;77(2):500–5.

10. Kaplan M, Cimen S, Kut MS, Demirtas MM. Cardiac Operations for Patients with Chronic Liver Disease. Heart Surg Forum. 2002;7.

11. Klemperer JD, Ko W, Krieger KH, Connolly M, Rosengart TK, Altorki NK, et al. Cardiac Operations in Patients With Cirrhosis. Ann Thorac Surg. 1998 Jan 1;65(1):85–7.

12. Komoda T, Frumkin A, Knosalla C, Hetzer R. Child-Pugh Score Predicts Survival After Radical Pericardiectomy for Constrictive Pericarditis. Ann Thorac Surg. 2013 Nov 1;96(5):1679–85.

13. Lin C-H, Hsu R-B. Cardiac surgery in patients with liver cirrhosis: Risk factors for predicting mortality. World J Gastroenterol. 2014 Sep 21;20(35):12608–14.

14. Lopez-Delgado JC, Esteve F, Javierre C, Perez X, Torrado H, Carrio ML, et al. Short-term independent mortality risk factors in patients with cirrhosis undergoing cardiac surgery. Interact Cardiovasc Thorac Surg. 2013 Mar 1;16(3):332–8.

15. Macaron C, Hanouneh IA, Suman A, Lopez R, Johnston D, Carey WW. Safety of Cardiac Surgery for Patients With Cirrhosis and Child–Pugh Scores Less Than 8. Clin Gastroenterol Hepatol. 2012 May 1;10(5):535–9.

16. Morisaki A, Hosono M, Sasaki Y, Kubo S, Hirai H, Suehiro S, et al. Risk Factor Analysis in Patients With Liver Cirrhosis Undergoing Cardiovascular Operations. Ann Thorac Surg. 2010 Mar 1;89(3):811–7.

17. Murashita T, Komiya T, Tamura N, Sakaguchi G, Kobayashi T, Furukawa T, et al. Preoperative evaluation of patients with liver cirrhosis undergoing open heart surgery. Gen Thorac Cardiovasc Surg. 2009 Jun 1;57(6):293–7.

18. Sugimura Y, Toyama M, Katoh M, Kato Y, Hisamoto K. Analysis of open heart surgery in patients with liver cirrhosis. Asian Cardiovasc Thorac Ann. 2012 Jun 1;20(3):263–8.

19. Suman A, Barnes DS, Zein NN, Levinthal GN, Connor JT, Carey WD. Predicting outcome after cardiac surgery in patients with cirrhosis: A comparison of Child-Pugh and MELD scores. Clin Gastroenterol Hepatol. 2004 Aug 1;2(8):719–23.

20. Yamane K, Izumi K, Yamachika S, Hashizume K, Tanigawa K, Miura T, et al. Operative Outcome of Cardiac Surgery in Patients with Liver Cirrhosis. :7.

21. Ailawadi G, LaPar DJ, Swenson BR, Siefert SA, Lau C, Kern JA, et al. Model for End-Stage Liver Disease Predicts Mortality for Tricuspid Valve Surgery. Ann Thorac Surg. 2009 May 1;87(5):1460–8.

22. Chokshi A, Cheema FH, Schaefle KJ, Jiang J, Collado E, Shahzad K, et al. Hepatic dysfunction and survival after orthotopic heart transplantation: Application of the MELD scoring system for outcome prediction. J Heart Lung Transplant. 2012 Jun 1;31(6):591–600.

23. Deo SV, Daly RC, Altarabsheh SE, Hasin T, Zhao Y, Shah IK, et al. Predictive Value of the Model for End-Stage Liver Disease Score in Patients Undergoing Left Ventricular Assist Device Implantation. ASAIO J. 2013 Feb;59(1):57–62.

24. Grimm JC, Shah AS, Magruder JT, Kilic A, Valero V, Dungan SP, et al. MELD-XI Score Predicts Early Mortality in Patients After Heart Transplantation. Ann Thorac Surg. 2015 Nov 1;100(5):1737–43.

25. Hawkins RB, Young BAC, Mehaffey JH, Speir AM, Quader MA, Rich JB, et al. Model for End-Stage Liver Disease Score Independently Predicts Mortality in Cardiac Surgery. Ann Thorac Surg. 2019 Jun 1;107(6):1713–9.

26. Loforte A, Fiorentino M, Gliozzi G, Mariani C, Folesani G, Suarez SM, et al. Heart Transplant and Hepato-Renal Dysfunction: The Model of End-Stage Liver Disease Excluding International Normalized Ratio as a Predictor of Postoperative Outcomes. Transplant Proc. 2019 Nov 1;51(9):2962–6.

27. Murata M, Kato TS, Kuwaki K, Yamamoto T, Dohi S, Amano A. Preoperative hepatic dysfunction could predict postoperative mortality and morbidity in patients undergoing cardiac surgery: Utilization of the MELD scoring system. Int J Cardiol. 2016 Jan 15;203:682–9.

28. Ortiz-Bautista C, García-Cosio MD, Lora-Pablos D, Ponz-de Antonio I, Rodríguez-Chaverri A, Morán-Fernández L, et al. Predicting Short- and Long-Term Outcomes in Adult Heart Transplantation: Clinical Utility of MELD-XI Score. Transplant Proc. 2018 Dec 1;50(10):3710–4.

29. Radakovic D, Opacic D, Börgermann J, Hsieh WC, Krutzinna M, Gummert J, et al. Model for end-stage liver disease predicts mortality after pericardiectomy for constrictive pericarditis†. Interact Cardiovasc Thorac Surg. 2018 Dec 1;27(6):813–8.

30. Tsuda K, Koide M, Kunii Y, Watanabe K, Miyairi S, Ohashi Y, et al. Simplified model for end-stage liver disease score predicts mortality for tricuspid valve surgery†. Interact Cardiovasc Thorac Surg. 2013 May 1;16(5):630–5.

31. Woolley JR, Kormos RL, Teuteberg JJ, Bermudez CA, Bhama JK, Lockard KL, et al. Preoperative liver dysfunction influences blood product administration and alterations in circulating haemostatic markers following ventricular assist device implantation. Eur J Cardiothorac Surg. 2015 Mar 1;47(3):497–504.

32. Yalcin YC, Muslem R, Veen KM, Soliman OI, Manintveld OC, Darwish Murad S, et al. Impact of preoperative liver dysfunction on outcomes in patients with left ventricular assist devices. Eur J Cardiothorac Surg. 2020 May 1;57(5):920–8.

33. Yang JA, Kato TS, Shulman BP, Takayama H, Farr M, Jorde UP, et al. Liver dysfunction as a predictor of outcomes in patients with advanced heart failure requiring ventricular assist device support: Use of the Model of End-stage Liver Disease (MELD) and MELD eXcluding INR (MELD-XI) scoring system. J Heart Lung Transplant. 2012 Jun 1;31(6):601–10.
